# Inhibition of Cyclic Adenosine Monophosphate-Specific Phosphodiesterase by Various Food Plant-Derived Phytotherapeutic Agents

**DOI:** 10.3390/medicines4040080

**Published:** 2017-11-04

**Authors:** Teresa Röhrig, Olga Pacjuk, Silvia Hernández-Huguet, Johanna Körner, Katharina Scherer, Elke Richling

**Affiliations:** Division of Food Chemistry and Toxicology, University of Kaiserslautern, Erwin-Schroedinger-Straße 52, 67663 Kaiserslautern, Germany; roehrig@chemie.uni-kl.de (T.R.); olga_pacjuk_@freenet.de (O.P.); siviahh_20_bcn@hotmail.com (S.H.-H.); johanna-koerner@gmx.de (J.K.); katha-scherer@gmx.de (K.S.)

**Keywords:** *Arbutus unedo*, *Camellia sinensis*, *Cynara scolymus*, *Zingiber officinale*, phosphodiesterase, cyclic AMP

## Abstract

**Background:** Phosphodiesterases (PDEs) play a major role in the regulation of cyclic adenosine monophosphate (cAMP)- and cyclic guanosine monophosphate (cGMP)-mediated pathways. Their inhibitors exhibit anti-inflammatory, vasodilatory and antithrombotic effects. Therefore, consumption of foods with PDE-inhibiting potential may possess beneficial influence on the risk of cardiovascular diseases. **Methods:** Four plant extracts (*Arbutus unedo*, *Camellia sinensis*, *Cynara scolymus*, *Zingiber officinale*) with promising ingredient profiles and physiological effects were tested for their ability to inhibit cAMP-specific PDE in vitro in a radioactive assay. **Results:** Strawberry tree fruit (*Arbutus unedo*) and tea (*Camellia sinensis*) extracts did not inhibit PDE markedly. Alternatively, artichoke (*Cynara scolymus*) extract had a significant inhibitory influence on PDE activity (IC_50_ = 0.9 ± 0.1 mg/mL) as well as its flavone luteolin (IC_50_ = 41 ± 10 μM) and 3,4-dicaffeoylquinic acid (IC_50_ > 1.0 mM). Additionally, the ginger (*Zingiber officinale*) extract and one of its constituents, [6]-gingerol, significantly inhibited PDE (IC_50_ = 1.7 ± 0.2 mg/mL and IC_50_ > 1.7 mM, respectively). Crude fractionation of ginger extract showed that substances responsible for PDE inhibition were in the lipoid fraction (IC_50_ = 455 ± 19 μg/mL). **Conclusions:** A PDE-inhibitory effect was shown for artichoke and ginger extract. Whether PDE inhibition in vivo can be achieved through ingestion of artichoke or ginger extracts leading to physiological effects concerning cardiovascular health should be addressed in future research.

## 1. Introduction

Phosphodiesterases (PDEs) hydrolyze phosphodiester bonds in the second messengers 3′,5′-cyclic adenosine monophosphate (cAMP) and 3′,5′-cyclic guanosine monophosphate (cGMP), resulting in the nucleoside 5′-monophosphates AMP and GMP, respectively. Along with adenylate and guanylate cyclases, PDEs play a major role in regulating cAMP- and cGMP-mediated signaling. Thus far, 11 PDE families, differing in substrate specificity and inhibitor selectivity, have been identified. Molecules that can selectively inhibit PDEs are attractive for pharmaceutical research because the inhibition of PDEs results in various physiological effects [1,2]. To demonstrate, PDE V inhibitors are used to treat hypertension because cGMP activates nitric oxide-mediated vasodilation [3]. Furthermore, PDE III and V inhibitors show significant antithrombotic effects through the inhibition of thrombin-mediated platelet activation [2]. Moreover, the nonspecific PDE inhibitor theophylline has anti-inflammatory properties and is used as additional therapy for patients with asthma or chronic obstructive pulmonary disease (COPD) [4]. These add-on therapy approaches can also use specific inhibitors, such as the newly licensed PDE IV inhibitor roflumilast, which is used to treat COPD [5]. However, certain compounds originating from foods can inhibit PDE. Caffeine and other methylxanthines, for example, had already been recognized as nonspecific inhibitors at the time of phosphodiesterase discovery [6,7]. Coffee has been shown to have both in-vitro and in-vivo PDE-inhibiting effects [8,9,10]. Furthermore, some classes of flavonoids (flavonols, flavones, anthocyanins and anthocyanidins, flavanones, and flavanonols) have shown PDE-inhibiting potential, whereas catechins are not potent PDE inhibitors [11,12,13,14,15]. Foods containing flavonoids have also demonstrated potent PDE inhibition, as shown by the in-vitro inhibition of cGMP-specific PDEs by red grape extract [16]. These results have led to a hypothesis that food compounds with PDE-inhibitory properties could lead to a PDE inhibition in vivo resulting in physiological effects with beneficial impact on health. Furthermore, certain plants that are traditionally used in phytotherapy to prevent, and treat, ischemic and inflammatory diseases, are also consumed as food. However, for most of these foods only parts of the mechanism of action are known and the efficacy of these compounds is still under discussion. To connect PDE inhibition with their physiological effects, we tested the PDE inhibitory potential of four promising food plant extracts: strawberry tree (*Arbutus unedo* L., Ericaceae) fruit extract (SFE), green tea (*Camellia sinensis* L., Theaceae) leaf extract (TXE), artichoke (*Cynara scolymus* L., Asteraceae) leaf extract (ALE) and ginger (*Zingiber officinale* Roscoe, Zingiberaceae) rhizome extract (GPE).

*Arbutus unedo* is used as an alternative treatment for hypertension and diabetes in Morocco [17]. It has antihypertensive effects, which have been attributed to a nitric oxide-dependent mechanism that is still not completely understood [18,19]. Furthermore, extracts from *Arbutus unedo* leaves have been shown to have anti-aggregative effects, as they significantly reduce in-vitro thrombin-induced platelet aggregation in a concentration-dependent manner [20,21]. These effects suggest PDE inhibition as a possible mechanism of action. The edible berries are used to produce jams, liqueurs and beverages, but these are only distributed regionally throughout the Mediterranean countries. The fruits are rich in gallic acid derivatives, proanthocyanidins, catechins and anthocyanins [22,23,24]. The fresh fruit contains anthocyanins at concentrations between 0.5 and 9.7 mg per 100 g, with cyanidin-3-*O*-galactoside as the most abundant anthocyanin present [22,23,24]. The well-known hydroquinone derivative arbutin (hydroquinone β-d-glucopyranoside) can also be extracted from both the leaves and fruits of *Arbutus unedo* [23,25]. The fruits also contain small amounts of flavonols and ellagic acid derivatives [22].

*Camellia sinensis* leaves are the base for different kinds of teas, which are classified by fermentation stage. Green, unfermented tea has the highest flavonoid content among different tea types, especially in terms of catechins, ranging from approximately 8% to 30% of dry matter. Teas contain various catechins, such as (−)-epigallocatechin gallate (EGCG), (−)-epicatechin gallate (ECG), (−)-epigallocatechin (EGC), (−)-epicatechin (EC), (−)-gallocatechin gallate (GCG), (−)-gallocatechin (GC) and (+)-catechin (C), with EGCG being the most abundant [26,27,28,29,30,31,32]. The caffeine content of green tea leaves normally averages between 2% and 4% of dry matter [26,28,31,33], but values up to almost 8% have been reported, with theobromine (<0.6%) and theophylline (<0.06%) appearing in insignificant amounts [30]. Green tea can have various physiological effects through antioxidant, anti-inflammatory and antithrombotic properties, and its consumption has been suggested to decrease the risk of degenerative diseases, especially cardiovascular disease [34,35]. The antithrombotic effect of green tea results from catechin-mediated inhibition of platelet activation. The mechanism of this antiplatelet activity is still not fully understood, but is correlated with increased intracellular cAMP levels [36,37,38]. Together with the ingredient profile, these physiological effects point to PDE inhibition as a possible mechanism of action.

The flower buds, prior to blooming, of *Cynara scolymus* (artichoke), are relevant for culinary purposes, while the leaves are processed for phytotherapeutic applications. Extracts from artichoke leaves are used in the prevention of arteriosclerosis due to cholesterol-lowering [39,40] and antihypertensive effects [41]. The antihypertensive effect results from an upregulation of endothelial-type nitric oxide synthase genes [41]. Furthermore, artichokes have antioxidant properties as they are rich in phenolic compounds, such as chlorogenic acids and flavones [42,43]. Chlorogenic acids can be found in artichoke heads at levels reaching 9.4 g/kg dry matter, with 5-caffeoylquinic acid and 1,5-dicaffeoylquinic acid the most prominent mono- and dicaffeoylquinic acids, respectively. 1,3-dicaffeoylquinic acid (cynarin) can also be found in artichokes in smaller amounts, and is mostly formed through heat-induced isomerization. The most prominent flavones include luteolin (concentrations reaching 0.7 g/kg dry matter) and apigenin (concentrations reaching 5.4 g/kg dry matter), which are mainly present as glucuronides, but exist also as glucosides and rutinosides. To contrast, the amounts of the flavanones naringenin 7-*O*-glucoside and narirutin in artichokes are insignificant, and aglycones are only found in trace amounts [44,45,46]. Phenolic compound concentrations are even higher in artichoke leaves than in the globe heads [47]. Furthermore, sesquiterpene lactones, specifically guaianolides, have been identified in *Cynara scolymus* [48]. Rich in flavonoids, artichokes may possess PDE-inhibiting potential.

Ginger (*Zingiber officinale* Roscoe), especially the rhizome, is commonly used in phytotherapy and has a long history in traditional Chinese medicine (TCM). Furthermore, it is a key ingredient of Asian cuisine. Its use in European cuisine is also on a steady rise. The net import of ginger and ginger products into Europe in 2012 was estimated at 59,000 tons, around 35,000 tons more than in 2002 (FAOSTAT). The constituents responsible for ginger’s pungent taste and rich aroma are found in non-volatile and volatile fractions of rhizome oleoresin. The non-volatile fraction contains a group of pungent compounds that contain a phenolic ketone moiety. These phenolic compounds include gingerols and their degradation products: shogaols, gingerdiols, gingerdiones, dehydrogingerdiones, diacetoxy gingerdiols, acetoxy gingerols, acetoxy gingerdiols and paradols [49]. The most abundant phenolic compound in fresh rhizome is [6]-gingerol, with concentrations ranging between 120–2100 μg/g [50]. The physiological effects, and therefore applications, of ginger in phytotherapy are numerous, ranging from anti-emetic and antidiabetic therapy (serotonin antagonism) [51,52,53,54,55] to analgesic, anti-inflammatory and antithrombotic effects (cyclooxygenase inhibition) [56]. Furthermore, the phenolic compounds found in ginger, especially dehydrogingerdiones, are potent antioxidants [57,58,59]. The abundancy of secondary plant metabolites and the multiple physiological effects of ginger indicate PDE inhibition as a possible mechanism of action.

These four food plants may contain potent PDE inhibitors explaining their physiological effects. To investigate this, extracts of these four food plants were tested for their inhibiting potential on cAMP-specific PDE in vitro, with the aim to then identify the compounds responsible for PDE inhibition and quantify their effectiveness as inhibitors. *Arbutus unedo*, *Camellia sinensis* and *Cynara scolymus* have not been tested for PDE inhibitory potential before. However, several studies have researched the PDE inhibitory potential of *Zingiber officinale*, but the results are inconsistent [60,61,62,63].

## 2. Materials and Methods

### 2.1. Chemicals, Cell Lines and Reagents

All the chemicals and reagents used were purchased in analytical quality. The purity of reference substances was >95%. 3,4-dicaffeoylquinic acid (3,4-DiCQA) was available from earlier studies [64]. The AMP, arbutin, benzamidine, bovine serum albumin (BSA), caffeine, cAMP, formic acid, [6]-gingerol, luteolin-7-*O*-glucuronide, 3,4,5-trimethoxycinnamic acid and XAD16N materials were purchased from Sigma Aldrich (Taufkirchen, Germany); apigenin, luteolin and luteolin-7-*O*-glucoside from Extrasynthese (Genay Cedex, France); leupeptin, pepstatin A and phenylmethylsulfonyl fluoride from Alexis Biochemicals (Loerrach, Germany); rolipram from Calbiochem (La Jolla, CA, USA); and [2,8-^3^H]-3′5′-cyclic adenosine monophosphate ammonium salt, 9.25 MBq/mL, from Hartmann Analytic (Braunschweig, Germany). The LXFL529L cell line was kindly provided by Prof. Fiebig (Freiburg, Germany).

### 2.2. Strawberry Tree Fruit Extraction 

*Arbutus unedo* L., Ericaceae, fruits were collected in the Najac region of Languedoc, Southern France during October 2014. A sample of 91 g of fruit was homogenized and extracted with H_2_O/methanol/formic acid 38/60/2 (*v*/*v*/*v*) for 4 h. Briefly after paper filtration (MN 615, Macherey Nagel, Düren, Germany) and partial concentration, the crude extract was purified with an XAD16N-packed column (50 mL). The column had been equilibrated with 500 mL 2% formic acid, and after applying 33 mL of the crude extract the column was washed with 300 mL 2% formic acid. Elution was carried out with 300 mL 90% ethanol. The eluate was dried in a vacuum and the residue was freeze-dried and homogenized. The yield of strawberry tree fruit extract (SFE) from the sample was 0.8%.

### 2.3. Quantification of Arbutin in SFE

Arbutin quantification in SFE was performed according to [65], with slight modifications, on an Agilent 1200 series HPLC system (Model G1312B) (Agilent Technologies, Santa Clara, CA, USA) equipped with a degasser (G1379B), binary pump (G1312B), auto-sampler (G1317C), column oven (G1316B) and DAD detector (G1315). The HPLC conditions: column Luna 5 μm C18 100 Å, 250 × 4.6 mm (Phenomenex, Torrance, CA, USA); solvent system: A—0.04% formic acid, B—methanol; gradient profile: isocratic 2% B for 3 min, 25% B over 1 min, isocratic 25% B for 4 min, 50% B over 5 min, 80% B over 10 min, isocratic 80% B for 7 min, 2% B over 2 min, isocratic 2% B for 5 min; flow rate: 0.6 mL/min; injection volume: 20 μL; sample concentration: 1 mg/mL in 50% methanol; UV-detection: λ = 280 nm; internal standard: caffeine (10 μg/mL). LOD and LOQ were identified with LOD = 0.1 μg/mL and LOQ = 0.5 μg/mL.

### 2.4. Green Tea Extraction (TXE)

*Camellia sinensis* L., Theaceae, plant material was purchased from a local food market as dried, green tea leaves “gunpowder” (“Bio Grüntee Gunpowder”, Alnatura, Bickenbach, Germany, LOT 65651). A sample of 18.04 g of the dried tea leaves was ground and extracted with 300 mL boiling water (95 °C) for 5 min with constant stirring. The mixture was cooled to room temperature, filtered (MN 615, Macherey Nagel, Düren, Germany), and 100 mL was purified with a XAD16N-packed column (50 mL). The column had been equilibrated with 500 mL water. Fifty milliliters of the mixture were applied and washed with 300 mL water, then eluted three times with 150 mL 90% ethanol. The eluate was dried in vacuum, freeze-dried and homogenized. The yield of green tea extract (TXE) from the sample was 4.4%. 

### 2.5. Quantification of Caffeine in TXE

The quantification of caffeine in the green tea extract (TXE) was performed with an Agilent 1200 series HPLC system (Model G1312B). HPLC conditions: column Synergi 4 μm Polar-RP 80 Å, 250 × 4.6 mm (Phenomenex); solvent system: A—0.1% formic acid, B—acetonitrile; gradient profile: 2%–12% B over 5 min, 12%–30% B over 15 min, 30%–90% B over 3 min, isocratic 90% B for 8 min, 90%–2% over 1 min, isocratic 2% B for 3 min; flow rate: 0.8 mL/min; column temperature: 40 °C; injection volume: 20 μL; sample concentration: 1 mg/mL in 50% methanol; UV-detection: λ–270, 325 nm; internal standard: 3,4,5-trimethoxycinnamic acid (20 μg/mL). LOD and LOQ were identified with LOD = 0.04 μg/mL and LOQ = 0.1 μg/mL.

### 2.6. Identification of Catechins in TXE

The catechins in the green tea extract were identified with a Perkin Elmer 200 series HPLC–UV (PerkinElmer, Waltham, MA, USA) equipped with a degasser, two micro pumps, an autosampler, and UV detector (785A) coupled to a PE Sciex API 2000 triple quad mass spectrometer (Sciex, Framingham, MA, USA). The HPLC conditions were partly adapted from [32]; HPLC conditions: column Luna 5 μm C18 100 Å, 250 × 4.6 mm (Phenomenex); column temperature: 40 °C; solvent system: A—H_2_O/methanol/formic acid: 74.7/25/0.3 (*v*/*v*/*v*), B—acetonitrile/formic acid: 99.7/0.3 (*v*/*v*); gradient profile: isocratic 0% B for 8 min, 100% B over 24 min, isocratic 100% B for 6 min, 0% B over 4 min, isocratic 0% B for 6 min; flow rate: 0.5 mL/min; injection volume: 20 μL; sample concentration: 1 mg/mL in 50% methanol; UV-detection: λ = 270 nm. ESI–MS/MS conditions: positive ion mode; ion spray voltage: 5500 V; temperature 450 °C; declustering potential: 61 V; focusing potential: 370 V; entrance potential: 12 V; collision cell entrance potential: 14 V.

### 2.7. Artichoke Leaf Extraction (ALE)

*Cynara scolymus* L., Asteraceae, plant material was purchased from a local pharmacy as dried and chopped leaves (Redwood, Bamberg, Germany, LOT 01160-033). A 12.3 g sample of leaves was homogenized and extracted with 300 mL 60% methanol for 3 h with stirring. After filtration (MN 615, Macherey Nagel, Düren, Germany), the extract was dried in a vacuum, freeze-dried and homogenized. This procedure yielded 26.8% of artichoke leaf extract (ALE).

### 2.8. Identification of Chlorogenic Acids and Flavones in ALE

The flavones and chlorogenic acids in the artichoke leaf extract were identified with a Perkin Elmer 200 series HPLC–UV coupled to a PE Sciex API 2000 triple quad electrospray mass spectrometer. HPLC conditions were the same as in the HPLC–UV analysis of caffeine in the TXE. ESI–MS/MS conditions: positive ion mode; ion spray voltage: 4700 V; temperature 450 °C; declustering potential: 50 V; focusing potential: 340 V; entrance potential: 10 V; collision cell entrance potential: 19 V; and for luteolin, declustering potential: 139 V; focusing potential: 129 V; entrance potential: 7 V.

### 2.9. Quantification of Dicaffeoylquinic Acids in ALE

The HPLC–UV analysis of the dicaffeoylquinic acids in the artichoke leaf extract was performed on an Agilent 1200 series HPLC system. The HPLC conditions for the quantification of an unidentified dicaffeoylquinic acid as 3,4-dicaffeoylquinic acid were the same as in the HPLC–UV analysis of the TXE. Internal standard: caffeine (10 μg/mL). LOD and LOQ were identified with LOD = 0.04 μg/mL and LOQ = 0.1 μg/mL.

### 2.10. Quantification of Flavones in ALE

The HPLC–UV analysis of the flavones in the artichoke leaf extract was performed on an Agilent 1200 series HPLC system; HPLC conditions: column Luna 5 μm C18 100 Å, 250 × 4.6 mm (Phenomenex); column temperature: 40 °C; solvent system: A—H_2_O/methanol/formic acid: 74.7/25/0.3 (*v*/*v*/*v*), B—acetonitrile/formic acid: 99.7/0.3 (*v*/*v*); gradient profile: isocratic 0% B for 8 min, 100% B over 30 min, isocratic 100% B for 6 min, 0% B over 4 min, isocratic 0% B for 6 min; flow rate: 0.5 mL/min; injection volume: 50 μL; sample concentration: 1 mg/mL in 50% methanol; UV-detection: λ = 270, 347 nm; internal standard: caffeine (10 μg/mL). LOD and LOQ were identified with LOD = 0.08 μg/mL and LOQ = 0.3 μg/mL.

### 2.11. Ginger Extraction and Fractionation 

*Zingiber officinale* Roscoe, Zingiberaceae, plant material was obtained from a local food market as commercially available dried and ground rhizome (Ostmann, Dissen am Teutoburger Wald, Germany, LOT 3326AB) A 6.0 g sample of the powder was extracted with 200 mL of water for 1 h, after which it was filtered (MN 615, Macherey Nagel, Düren, Germany) and freeze-dried. The yield of ginger powder extract (GPE) was 26.7%, of which 530 mg of this extract was reconstituted with 50 mL of H_2_O and the pH was adjusted to 3.0 with formic acid. The mixture was extracted three times with 50 mL ethyl acetate. The hydrophilic, ‘watery’ phase (GWE) was freeze-dried. The lipoid, organic phase (GLE) was dried over NaSO_4_ and then further dried in a vacuum. Weight comparison revealed that the GPE yielded 5% of the organic fraction (GLE) and 95% of the hydrophilic fraction (GWE).

### 2.12. Quantification of [6]-Gingerol in Ginger Extracts (GPE, GLE, GWE)

Quantification of [6]-gingerol from the three ginger extracts (GLE, GPE, GWE) followed the procedure of [66] on an Agilent 1200 series HPLC system (see above); HPLC conditions: column Symmetry 5 μm C18 100 Å, 4 μm 250 × 4.6 mm (Waters, Milford, MA); solvent system: A—0.1% formic acid, B—acetonitrile; gradient profile: 45%–50% B over 12 min, 50%–65% B over 13 min, 65%–100% B over 20 min, isocratic 100% B for 10 min, 100%–45% over 5 min, isocratic 45% B for 10 min; flow rate: 0.4 mL/min; injection volume: 8 μL; sample concentration: 1 mg/mL in methanol; UV-detection: λ = 230 nm, internal standard: 3,4,5-trimethoxycinnamic acid (11.8 μg/mL). LOD and LOQ were identified with LOD = 0.3 μg/mL and LOQ = 0.9 μg/mL.

### 2.13. Cell Culture

The large cell lung tumor xenograft cell line LXFL529L was cultivated in RPMI 1640 medium with 10% fetal calf serum (FCS) and 1% penicillin/streptomycin at 37 °C, 5% CO_2_ in humidified incubators. Medium, antibiotics and FCS were purchased from Gibco, Life Technologies, Carlsbad, CA, USA. Absence of mycoplasma contamination was verified regularly.

### 2.14. cAMP-Specific PDE Activity Assay

Phosphodiesterases were isolated from LXFL529L cells. Appearing in the particulate and cytosol of this cell line, cAMP hydrolyzing activity belongs mostly to PDE family 4 [67]. Cells were cultivated for 48 h, 10^6^ cells per culture dish, and then washed with 2 × 1 mL phosphate buffered saline (pH = 7.4, 4 °C) and harvested with 2 × 200 μL RUN III buffer (100 mM Tris/HCl 7.4, 20 mM MgCl_2_, 0.2 mM EDTA, 10 mM benzamidine, 1 mM β-mercaptoethanol and a protease inhibitor mix). The harvested cells underwent ultrasound lysis, after which the supernatant (12,000 rcf, 15 min, 4 °C), which contains the cytosolic phosphodiesterases, was collected and diluted with RUN III buffer to 4.5 mL. The inhibitory effects of the different extracts on PDE were measured according to Montoya et al. [8]. Fifty microliters of the cytosolic fraction, 50 μL of the sample and 50 μL of cAMP Mix (30 mM Tris/HCl pH 7.4, 9 mM MgCl_2_, 3 mM 5′AMP, 3 μM cAMP, 2.6 μCi/mL [2,8-^3^H]-cAMP) were mixed over ice and incubated for 10 min at 37 °C. The reaction was then stopped over ice, and 250 μL of ZnSO_4_ (0.266 M) and Ba(OH)_2_ (0.266 M) were added to the mixture. Then, 450 μL of the supernatant was mixed with 4 mL of a scintillation cocktail and the resulting radioactivity was measured with a liquid scintillation counter (TRI-CARB 2100 TR, Packard, Meriden, CT, USA). Samples were dissolved in either water or DMSO, which did not exceed a final concentration of 1% or 10%, respectively. The selective PDE 4 inhibitor rolipram (10 μM) served as a positive control. To compare with previous experiments, test concentrations of extracts and single substances were chosen in a similar range of previous studies (extracts <10 mg/mL, single substances <500 μg/mL) [10,68].

### 2.15. Statistical Analysis

The results of cAMP-specific PDE activity assay were presented as the mean ± standard deviation of at least three independent experiments. Statistical analyses were carried out with Excel 2013 software. The differences between samples and solvent control were evaluated by analysis of variance (one-side Fisher’s *F*-test, confidence interval *p* < 0.05) followed by a one-sided Student’s *t*-test. The following confidence intervals are represented as either one, two, or three stars in the figures: *p* < 0.05: *; *p* < 0.01: **; *p* < 0.001: ***.

## 3. Results

The extracts of strawberry tree (*Arbutus unedo* L., Ericaceae) fruits (SFE), green tea (*Camellia sinensis* L., Theaceae) leaves (TXE), artichoke (*Cynara scolymus* L., Asteraceae) leaves (ALE) and ginger (*Zingiber officinale* Roscoe, Zingiberaceae) rhizome (GPE) were tested for their in-vitro ability to inhibit cAMP-specific PDE activity in LXFL529L cells expressing mostly PDE IV isoforms. The inhibitory potentials of the extracts were compared to the selective PDE IV inhibitor rolipram. Data are presented as T/C (test over control) in percent of a solvent control.

### 3.1. Strawberry Tree Fruit Extraction (SFE) and Inhibitory Effect on PDE Activity In Vitro

**Figure 1:**
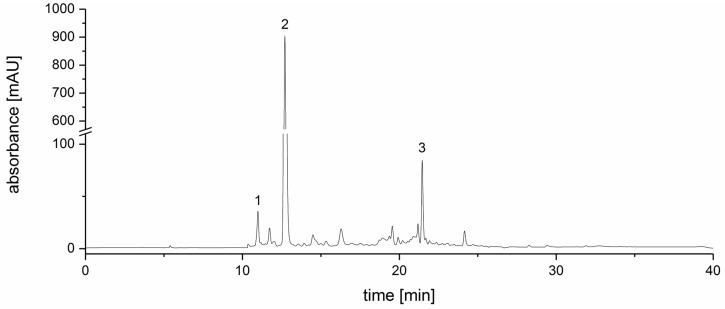
HPLC–UV chromatogram (λ = 280 nm) of the strawberry tree fruit extract (SFE) (*Arbutus unedo*). The three peaks represent arbutin (1), galloyl quinic acid (2) and the internal standard caffeine (3).

Extraction of the strawberry tree (*Arbutus unedo*) fruits followed by purification with a XAD16N column yielded less than 1% of polyphenol-enriched SFE as a red-colored, amorphous and hydrophobic powder that contained a total arbutin concentration of 20.0 ± 1.3 μg/mg. A chromatogram of the SFE is shown in Figure 1.

**Figure 2:**
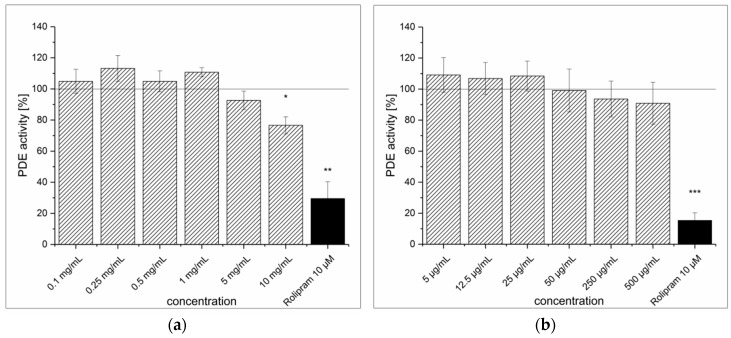
cAMP-specific phosphodiesterase (PDE) activity following incubation with strawberry tree fruit extract (SFE) (**a**) and purified arbutin (**b**). Data are presented as the mean ± standard deviation of three independent experiments. The significance of differences between sample and control was assessed with a Student’s *t*-test. Confidence intervals: * *p* < 0.05; ** *p* < 0.01; *** *p* < 0.001.

The SFE showed no in-vitro inhibitory effect on PDE at levels below 5 mg/mL (see Figure 2a). However, when the concentration was increased to 10 mg/mL, it showed a PDE inhibition of 23.6% (*p* < 0.05). When this inhibition was compared to that of the selective PDE IV inhibitor rolipram at a concentration of 10 μM (2.8 μg/mL, 70.5%), the effect was rather negligible. An IC_50_ value was not determined due to poor inhibition of the SFE (<50%). Furthermore, when PDE was incubated with pure arbutin, there was no inhibition at all in tested concentrations up to 500 μg/mL (1.8 mM) (Figure 2b).

### 3.2. Green Tea Leaves Extraction (TXE) and Inhibitory Effect on PDE Activity In Vitro

**Figure 3:**
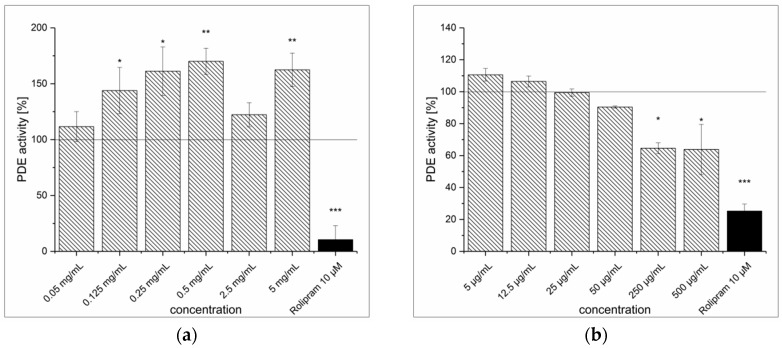
cAMP-specific phosphodiesterase (PDE) activity following incubation with green tea extract (TXE) (**a**) and caffeine (**b**). Data are presented as the mean ± standard deviation of three independent experiments. The significance of differences between sample and control was assessed with a Student’s *t*-test. Confidence intervals: * *p* < 0.05; ** *p* < 0.01; *** *p* < 0.001.

The extraction procedure of green tea leaves (*Camellia sinensis*) and purification of the polyphenol-enriched fraction with a XAD16N column yielded ca. 9% of TXE as a green-colored, amorphous and hydrophobic powder. The TXE did not inhibit PDE activity at any of the tested concentrations and while an inhibition was expected, it showed an increase of PDE activity (see Figure 3a). The content of caffeine, which is a well-known PDE inhibitor, in the TXE was 131.6 ± 5.0 μg/mg. The inhibitory effect of caffeine on PDE activity was concentration dependent and the maximum PDE inhibition of 40% was observed at concentrations of 250 μg/mL and 500 μg/mL (see Figure 3b). Earlier studies showed the IC_50_ for caffeine was determined to be 0.9 ± 0.1 mg/mL (4.8 ± 0.6 mM—data not shown [68]) under the same assay conditions.

### 3.3. Artichoke Leaves Extraction (ALE) and Inhibitory Effect on PDE Activity In Vitro

**Figure 4:**
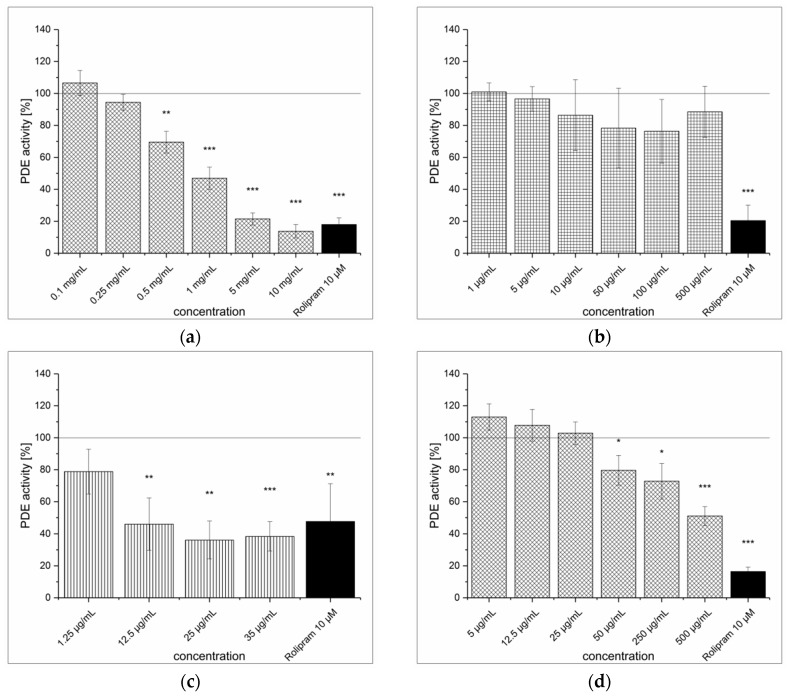
cAMP-specific phosphodiesterase (PDE) activity following incubation with artichoke leaf extract (ALE) (**a**), luteolin-7-*O*-glucuronide (**b**), luteolin (**c**) and 3,4-dicaffeoylquinic acid (**d**). Data are presented as the mean ± standard deviation of three independent experiments. The significance of differences between sample and control was assessed with a Student’s *t*-test. Confidence intervals: * *p* < 0.05; ** *p* < 0.01; *** *p* < 0.001.

The extraction of artichoke (*Cynara scolymus*) leaves yielded almost 27% of ALE as a brown-colored, amorphous powder. The ALE concentrations higher than 0.5 mg/mL showed significant inhibition of PDE activity (IC_50_ = 0.9 ± 0.1 mg/mL). At the highest tested concentrations (5 and 10 mg/mL), ALE showed levels of inhibition comparable to the positive control rolipram (see Figure 4a). The artichoke flavones luteolin, apigenin and luteolin-7-*O*-glucoside showed poor solubility (in 30% DMSO) and thus limited the concentrations to be tested. The highest concentrations of apigenin, luteolin-7-*O*-glucoside and luteolin-7-*O*-glucuronide used in the assay were 25 μg/mL, 35 μg/mL and 500 μg/mL, respectively, and none of these flavones showed any PDE inhibition. Data for luteolin-7-*O*-glucuronide are shown in Figure 4b. Conversely, it could be confirmed that unglycosylated luteolin acted as a strong PDE inhibitor at low concentrations (IC_50_ = 11.8 ± 2.9 μg/mL), shown in Figure 4c. Unfortunately, this flavone aglycone was not present in the extract. The predominant chlorogenic acid in the ALE was 5-caffeoylquinic acid (5-CQA), but a dicaffeoylquinic acid (DiCQA), where the positions of the esterification were not identified, was also present at 2.5 ± 0.5 μg/mg. The supposed ALE constituents 1,3-dicaffeoylquinic acid and 1,5-dicaffeoylquinic acid were not commercially available, so an isomer, 3,4-dicaffeoylquinic acid, was used for the PDE inhibition experiments. As 3,4-dicaffeoylquinic acid concentrations increased to 500 μg/mL (~ 1mM), PDE activity decreased almost 50% (see Figure 4d).

### 3.4. Dried Ginger Rhizome Extraction (GPE) and Inhibitory Effect on PDE Activity In Vitro

**Figure 5:**
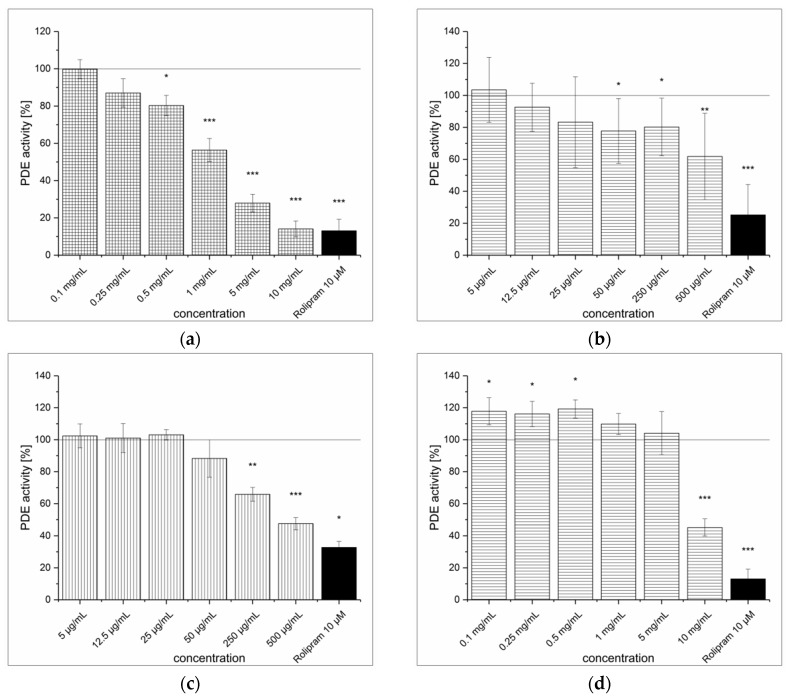
cAMP-specific phosphodiesterase (PDE) activity following incubation with ginger powder extract (GPE) (**a**), [6]-gingerol (**b**), ginger lipoid fraction (GLE) (**c**) and ginger hydrophilic fraction (GWE) (**d**). Data are presented as the mean ± standard deviation of three independent experiments. The significance of differences between sample and control was assessed with a Student’s *t*-test. Confidence intervals: * *p* < 0.05; ** *p* < 0.01; *** *p* < 0.001.

The extraction procedure of powdered ginger rhizome (*Zingiber officinale* Roscoe) resulted in a light brown, amorphous powder with a [6]-gingerol content of 10.0 ± 0.6 μg/mg. The GPE inhibited PDE activity significantly (see Figure 5a). At the highest tested concentration (10 mg/mL), the inhibitory effect of GPE (IC_50_ = 1.7 ± 0.2 mg/mL) was comparable to the positive control (85.9% vs. 86.9%). Furthermore, [6]-gingerol also had an inhibitory effect, as at a concentration of 500 μg/mL (~ 1.7 mM) it decreased PDE activity by 38.2% (see Figure 5b). Considering that [6]-gingerol content in GPE was 1%, the observed PDE inhibition cannot result exclusively from [6]-gingerol. A crude fractionation of the GPE with liquid–liquid extraction was performed to determine whether the PDE-inhibiting compounds were hydrophilic or lipophilic. The fractionation resulted in a lipoid (GLE) fraction that accounted for 5% and a hydrophilic (GWE) fraction that accounted for 95% of the GPE. Both fractions were able to inhibit PDE, but the lipoid fraction was a more potent inhibitor (IC_50_ = 455 ± 19 μg/mL) than the hydrophilic fraction (IC_50_ = 10.5 ± 1.9 mg/mL) (see Figure 5c,d). The [6]-gingerol content of the GLE fraction was 205.4 ± 11.7 μg/mg, whereas no gingerol was identified in the GWE fraction (LOD = 0.3 μg/mg). However, [6]-gingerol represented only 20% of GLE fraction, so the inhibition of PDE cannot be solely linked to [6]-gingerol.

## 4. Discussion

Both ginger extract GPE and artichoke extract ALE demonstrated strong PDE inhibitory potential in the frame of this study. Furthermore, PDE inhibition was observed with [6]-gingerol, the lead substance in ginger, as well as with luteolin, caffeine and 1,3-diCQA. Contrary to that, incubation with tea extract TXE, strawberry tree fruit extract SFE and arbutin did not result in PDE inhibition. 

Certain *Arbutus unedo* constituents, such as anthocyanins and catechins, have been reported to inhibit PDEs, although the inhibitory effect of catechins was shown to be marginal [11,12,13,14,15]. Anthocyanins are strong inhibitors of PDEs and, therefore, it was expected that the SFE would have a strong inhibitory effect on PDE. A plausible explanation for the lack of inhibition could be a low anthocyanin content in the SFE, which was not verified. Since the extraction was performed with 2% formic acid, which has been shown to be suitable in the extraction of anthocyanins [69], a loss of anthocyanins during extraction can be excluded. A low anthocyanin content in the SFE could partly result from the raw material. Previous reports have presented an unusually wide range for the anthocyanin content in fresh fruits, with values of 0.51 mg [23], 3.77 mg [22] and even 517.40 mg [70] per 100 g of fresh fruit. A possible explanation for this wide variation is that the chemical composition of the fruit changes at different stages of ripeness [71]. The antihypertensive [17,18,19] and antithrombotic [20,21] properties of *Arbutus unedo* indicate that compounds in the fruit could inhibit PDEs, but this study’s experiments with either the polyphenol-enriched extract SFE or arbutin were not able to confirm this. Fruits of *Arbutus unedo* are rich in biologically active compounds such as gallic acid derivatives, proanthocyanidins, anthocyanins and catechins, all of which can contribute to the reported effects mentioned above. However, PDE inhibition seems to not be a mechanism of action (MOA). Food that contain arbutin should be used with care, despite the traditional therapeutic use of plant-derived arbutin in urethral infections. Sometimes hydroquinone is released when arbutin undergoes deglycosylation by gut microbiota [72]. Hydroquinone is mutagenic and was assessed by the IARC as “not classifiable as to its carcinogenicity to humans (group 3)” due to inadequate evidence of carcinogenicity in humans.

As with SFE, it was hypothesized that *Camellia sinensis* extract TXE would inhibit PDE activity due to its chemical composition and recognized physiological effects, especially an antithrombotic effect [36,37,38]. It was expected that a TXE concentration of 5 mg/mL, with caffeine content of almost 0.7 mg/mL, would inhibit PDE activity, but this was not the case. This finding, together with the unusually high PDE activity, suggests that caffeine-mediated PDE inhibition in TXE is relatively weak, or may be diminished by an unknown PDE-activating mechanism. Furthermore, catechin and epicatechin have been shown to be weak PDE inhibitors when compared to other flavonoids [13,14]. Contrary to these findings, catechin and epicatechin were also found to have a stimulatory effect on PDEs with EC_50_ values of 240 and 136 μM, respectively [14]. The catechins identified in the TXE through HPLC–MS/MS were identical to those previously reported in literature [26,27,28,29,30,31,32,73,74], and their occurrence, based on extracted ion chromatogram area ratios, was EGC >> EC > EGCG ≈ GC > ECG > C. The total catechin content of the TXE was not determined in the frame of these investigations. As catechins have a weak inhibitory effect on PDEs, a plausible explanation for the absence of PDE inhibition by TXE may be low catechin concentrations in the TXE, possibly in combination with a PDE-activating mechanism that counteracts the inhibitory effect of caffeine. According to the TXE results, the physiological effects of *Camellia sinensis* cannot be explained by PDE inhibition.

Artichoke (*Cynara scolymus*) is known to contain flavones, mainly apigenin and luteolin glycosides [44,45,46], which have been reported to act as PDE inhibitors [12,13,14]. However, in this study no effect was observed when luteolin glycosides were tested. Furthermore, luteolin, which was demonstrated to be a strong PDE inhibitor in accordance with the literature, could not be found in the PDE-inhibiting extract. These circumstances make the interpretation of the PDE inhibition observed with artichoke extract ALE difficult. Chlorogenic acids are another group of phenolic compounds present in artichokes with physiological relevancy. Previous studies attribute part of the PDE inhibitory effect of the chlorogenic acid 5-CQA to a pH shift [8]. The involvement of pH change in PDE inhibition can be neglected as pH remained constant during assays with 3,4-DiCQA in these experiments. Hence, ALE showed a fairly strong inhibitory effect on PDE, possibly through dicaffeoylquinic acids and/or flavones that were not identified in the extract yet. The antihypertensive effect of *Cynara scolymus* [41] may result from PDE inhibition, but further investigations are necessary to confirm this role. As the content of phenolic compounds is higher in artichoke leaves than in the edible flower buds [47], the effect of consuming artichokes or drinking teas from artichoke leaves on PDE activity should be of relevance.

Earlier publications have not reported that ginger (*Zingiber officinale*) extracts, or [6]-gingerol, can inhibit PDE [60,61,62]. However, three potential PDE inhibitors from the Zingiberaceae family, one of which was from *Zingiber officinale,* were recently identified through in-silico screening [63]. These results provide further evidence that prepared ginger extract has the potential to inhibit PDE activity. A study where volunteers consumed different doses of a dietary ginger supplement found the C_max_ of [6]-gingerol metabolites reached a maximum of 4.4 μg/mL [75], while in this study IC_50_ values of the in-vitro PDE inhibition were 100-fold higher. This raises the question whether under physiological conditions a PDE-inhibiting effect from [6]-gingerol is likely. Nevertheless, ginger rhizome presumably contains further lipophilic PDE inhibitors with even higher potential than [6]-gingerol, as was concluded when the lipoid fraction demonstrated a stronger PDE-inhibiting potential than [6]-gingerol. Because these substances are unknown, their identification and pharmacokinetics are of strong interest. The inhibition of PDE by ginger extracts in vivo may have a synergistic effect on the physiological effects mediated by cyclooxygenase inhibition, but this should be investigated further.

The interpretation of results of this study faces some limitations due to the different approaches used for each of the four extracts. Unfortunately, some known PDE inhibitors were not quantified in the extracts showing no PDE inhibition, for example, anthocyanins in SFE and catechins in TXE. Thus, explaining why these extracts possessed no inhibitory effect on PDE activity with a low concentration of these PDE inhibitors in the extract is only hypothetical. Furthermore, the results for luteolin, 3,4-diCQA and [6]-gingerol showed that single-substance testing only led to vague conclusions about the active compounds in ALE and GPE. An activity-guided fractionation should therefore be preferred in contrast to single-substance testing to identify responsible compounds in PDE-inhibiting extracts. This may be reversed with simple screening studies.

## 5. Conclusions

This study was able to demonstrate the PDE-inhibiting potential of artichoke and ginger extracts for the first time. The motivation to investigate selected food extracts on their ability to inhibit PDE was triggered by previous reports of physiological effects that may result from PDE inhibition, for example, antithrombotic and antihypertensive effects. The results of this study are summarized in Table 1 Extracts from *Arbutus unedo* and *Camellia sinensis* did not show notable inhibition of PDE in our experiments, and thus the reported physiological effects of these plants do not result from underlying PDE inhibition. On the other hand, extract from artichoke (*Cynara scolymus*) showed significant PDE inhibition. The results of this study suggest that flavones and caffeoylquinic acids are involved in the PDE inhibition of ALE. Whether PDE inhibition is a mechanism of action in the reported physiological effects of artichoke should be addressed in future studies. Furthermore, extract from ginger (*Zingiber officinale*) also had an inhibitory effect on PDE activity. It is likely that, next to [6]-gingerol, certain unidentified lipophilic compounds are of relevance for the observed effect. It should be investigated in the future whether PDE inhibition can be achieved under physiological conditions with ginger extracts. Furthermore, the results should be validated, and possible beneficial in-vivo effects of artichoke and ginger are to be investigated.

## Figures and Tables

**Table 1:** Inhibition (IC_50_) of cAMP-specific phosphodiesterase (PDE) by the four extracts and their constituent compounds. The content values are presented as the mean ± standard deviation of two independent experiments. Percentages in brackets represent inhibition at highest concentration.

Extracted Plant	Extracted Part	IC_50_ (mg/mL)	Lead Substance	Content (μg/mg)	IC_50_	
*Arbutus unedo* (strawberry tree)	fruit	n.d. ^1^	arbutin	20.0 ± 1.3	n.d. ^1^	n.d. ^1^
*Camellia sinensis* (tea)	dried leaves	n.d. ^1^	caffeine	131.6 ± 5.0	0.9 ± 0.1 mg/mL	4.8 ± 0.6 mM
*Cynara scolymus* (artichoke)	dried leaves	0.9 ± 0.1	3,4-diCQA	2.5 ± 0.5	>500 μg/mL (49%)	>1.0 mM
			luteolin	<0.08	11.8 ± 2.9 μg/mL	41 ± 10 μM
			luteolin-7-*O*-glucuronide	2.7 ± 0.3	n.d. ^1^	n.d. ^1^
			luteolin-7-*O*-glucoside	4.9 ± 1.0	n.d. ^1^	n.d. ^1^
			apigenin	<0.08	n.d. ^1^	n.d. ^1^
*Zingiber officinale* (ginger)	dried rhizome	1.7 ± 0.2	[6]-gingerol	10.0 ± 0.6	>500 μg/mL (38%)	>1.7 mM
			lipoid fraction (GLE)	~50	455 ± 19 μg/mL	-
			hydrophilic fraction (GWE)	~950	10.5 ± 1.9 mg/mL	-

^1^ n.d.—not determined due to solubility limitations or weak inhibitory effect.
